# Clean air is the key to clean lungs: Secondhand smoke is injurious to health

**DOI:** 10.1186/1477-3163-5-7

**Published:** 2006-02-07

**Authors:** Gopala Kovvali

**Affiliations:** 1 Editor-in-Chief, Journal of Carcinogenesis, Founder President, Carcinogenesis Foundation, 22 Heritage Drive, Edison, NJ 08820, USA

On behalf of the editorial board of JC and my own behalf I wish the carcinogenesis research community a Happy and Healthy 2006.

Several publications in the Journal of Carcinogenesis and other related journals during 2005 shed light on cancer causing aspects and mechanisms of various diets and carcinogens present in them. I want to share three incidents that occurred in 2005 that made me think seriously.

In the beginning of 2005, a Chinese friend of mine told me with sadness that his sister had lung cancer and was undergoing a surgery. She was 53 years old. He was very anxious to know if surgery could help. She lives in China and never smoked a cigarette.

In the middle of July I heard that my friend's father had lung cancer. He was 60 years old. My immediate reaction was 'that is sad, does he smoke'. My friend told me that he smoked when he was young. Before I could digest that information, I heard of the death of my friend's father.

I got a call from my friend during early January 2006. After exchanging pleasantries and New Year greetings, I asked how his parents were whom I met couple of months ago. I was shocked to hear that his mother was diagnosed with lung cancer. I did not ask if she smoked as it is a rare habit among middle class traditional women in India. I recall the active and healthy looking person in her late 50's with whom I shared a table at a party not too long ago.

I am sure that these three incidents represent a fraction of many incidents of lung cancer among non-smokers. It is estimated that 15–20% of lung cancer patients around the world never smoked. What is the basis for the incidence of lung cancer in these patients? The obvious answer seems to be environmental carcinogens.

'Smoking is injurious to health'. We are all familiar with this statutory warning printed on cigarette packets. Unfortunately, what this warning does not convey is that smoking by you or by others is injurious to the health of communities. Several states in the United States have enacted clean air acts that ban cigarette smoking in public places like restaurants. New Jersey joined the league of these states in January 2006 and is the 11^th ^state of USA that enacted clean air act. This is a very important step in enhancing the quality of public health and reducing the economic burden on the states.

An accompanying Opinion 'The Case For Clean Indoor Air' by Dr. Fred M. Jacobs, the Commissioner of the Department of Health and Senior Services of the state of New Jersey, USA, presents very compelling statistics and arguments in favor of banning smoking in public places. In fact, he played a pivotal role in formulating and in having the clean air act passed in the New Jersey state Assembly. I hope health commissioners in the other states of USA and around the world do what Dr. Jacobs did- advice the governments and ensure that the governments recognize that people should have the freedom to breath clean air. Hopefully, the message 'Secondhand smoke is injurious to health' will soon be spread as a statutory warning.

